# Supplementary material for: Molecular basis of AKAP79 regulation by calmodulin
Source: Nat Commun. 2017 Nov 22;8:1681. doi: 10.1038/s41467-017-01715-w (PMC5698441; doi:10.1038/s41467-017-01715-w)
Supplement: Supplementary file 1 — Supplementary Information [file 41467_2017_1715_MOESM1_ESM.pdf]

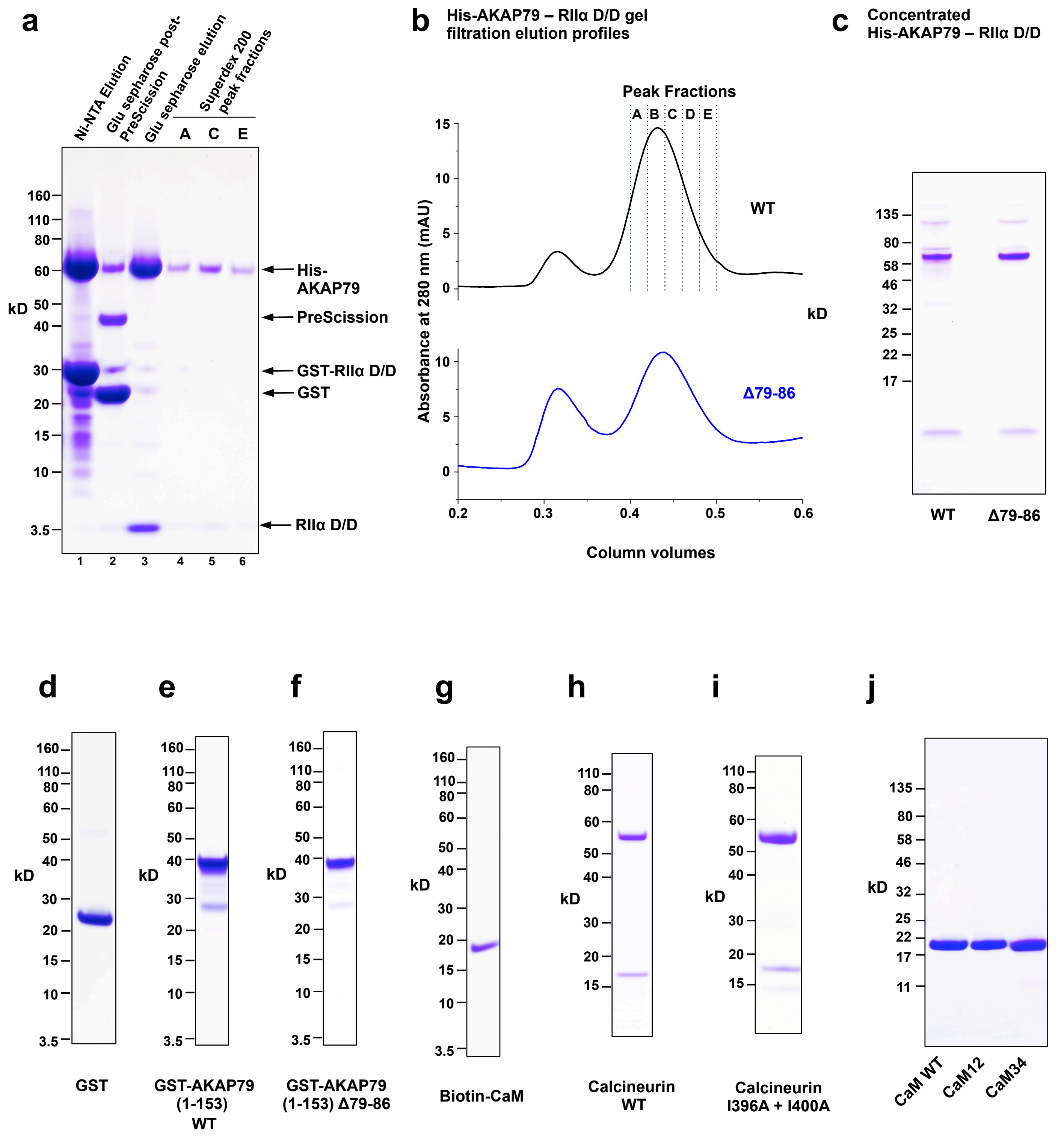

Supplementary Figure 1. **Preparation of purified proteins** (**a**) Coomassie-stained 4-12 % SDS-polyacrylamide gel showing sequential purification of His-AKAP79-D/D. The lanes show sample after elution from Ni-NTA agarose (lane 1), the bead (lane 2) and eluate (lane 3) fractions following incubation of glutathione sepharose beads with PreScission protease, and peak fractions eluted from a Superdex 200 column (lanes 4-6). (**b**) Relationship between 280 nm absorbance and elution volume for WT (upper trace) and Δ79-86 (lower trace) AKAP79-D/D in the final SEC purification step. Both proteins eluted with a peak volume of ~ 0.43 column volumes. (**c**) Coomassie gel showing purified WT and Δ79-86 AKAP79-D/D following concentration. (**d**)-(**j**) Coomassie-stained gels showing purity of the following proteins that were utilised during the investigation: GST (**d**), fusions of WT (**e**) and Δ79-86 (**f**) AKAP79 (1-153) at the C-terminus of GST, biotin-labelled CaM (**g**), WT (**h**) and I396A/I400A (**i**) calcineurin, and three variants of unlabeled CaM (**j**).

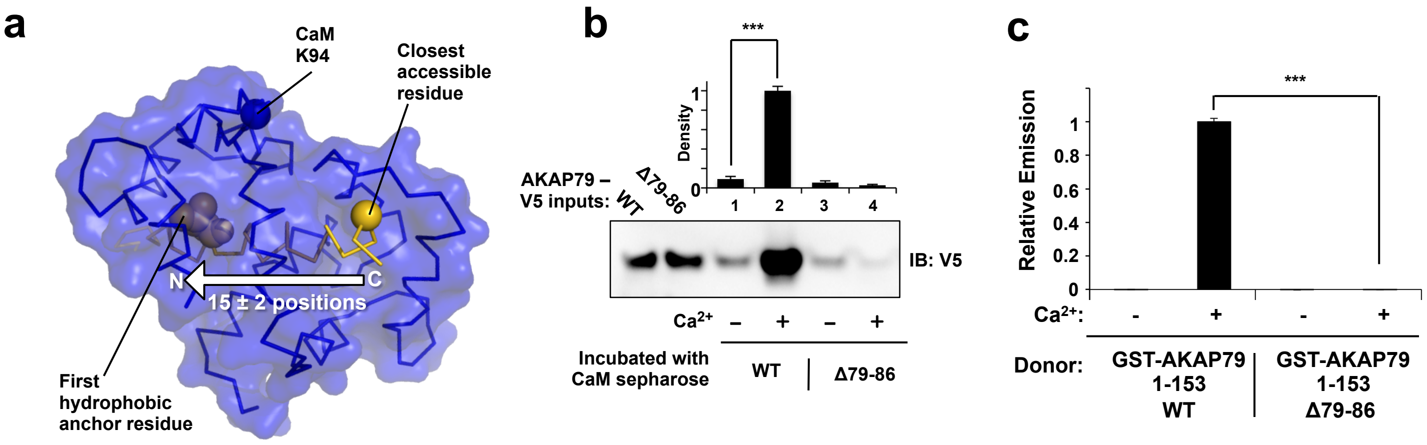

Supplementary Figure 2. Triangulation of the CaM binding site in AKAP79 (a) To predict where the CaM interaction helix in AKAP79 could fall on the basis of the XL-MS data, we analysed a representative selection of five Ca^{2+}/CaM - target complex structures (two 1-8-14 class motifs: 2LV6, 2JZI; two 1-5-10 class motifs: 2WEL, 1MXE; one atypical motif: 2KDU) with the aim of determining where $Lys94_{CaM}$ would be expected to crosslink most efficiently in each target in relation to the first hydrophobic anchor position. We calculated the distances of solvent-accessible amino acids in each target from $Lys94_{CaM}$ using NCONT. Amino acids were considered accessible when their accessible surface constituted > 0.4 of their total surface area. This analysis predicted that on average $K94_{CaM}$ would crosslink most efficiently at a position ~ 15 amino acids C-terminal to the first hydrophobic anchor position in the target's primary sequence, as shown in the cartoon. (**b**) Pull-down of either WT or Δ79-86 C-terminally V5-tagged full-length AKAP79 with CaM sepharose, with or without Ca^{2+}. AKAP79 pull-down was detected by anti-V5 immunoblotting (n=4). Ca^{2+} increased binding of WT AKAP79 by 11-fold ($p = 1.7 \times 10^{-5}$) according to densitometry, whereas the Δ79-86 variant did not bind efficiently irrespective of Ca^{2+}. (**c**) Alphascreen recordings of biotin-CaM association with WT or Δ79-86 GST-AKAP79 (1-153). Recordings were performed either with or without Ca^{2+} (n=6). Removal of amino acids 79-86 abolished interaction with CaM, according to this assay. ***$P < 0.001$ by two-tailed Student's t test. Error bars show s.e.m.

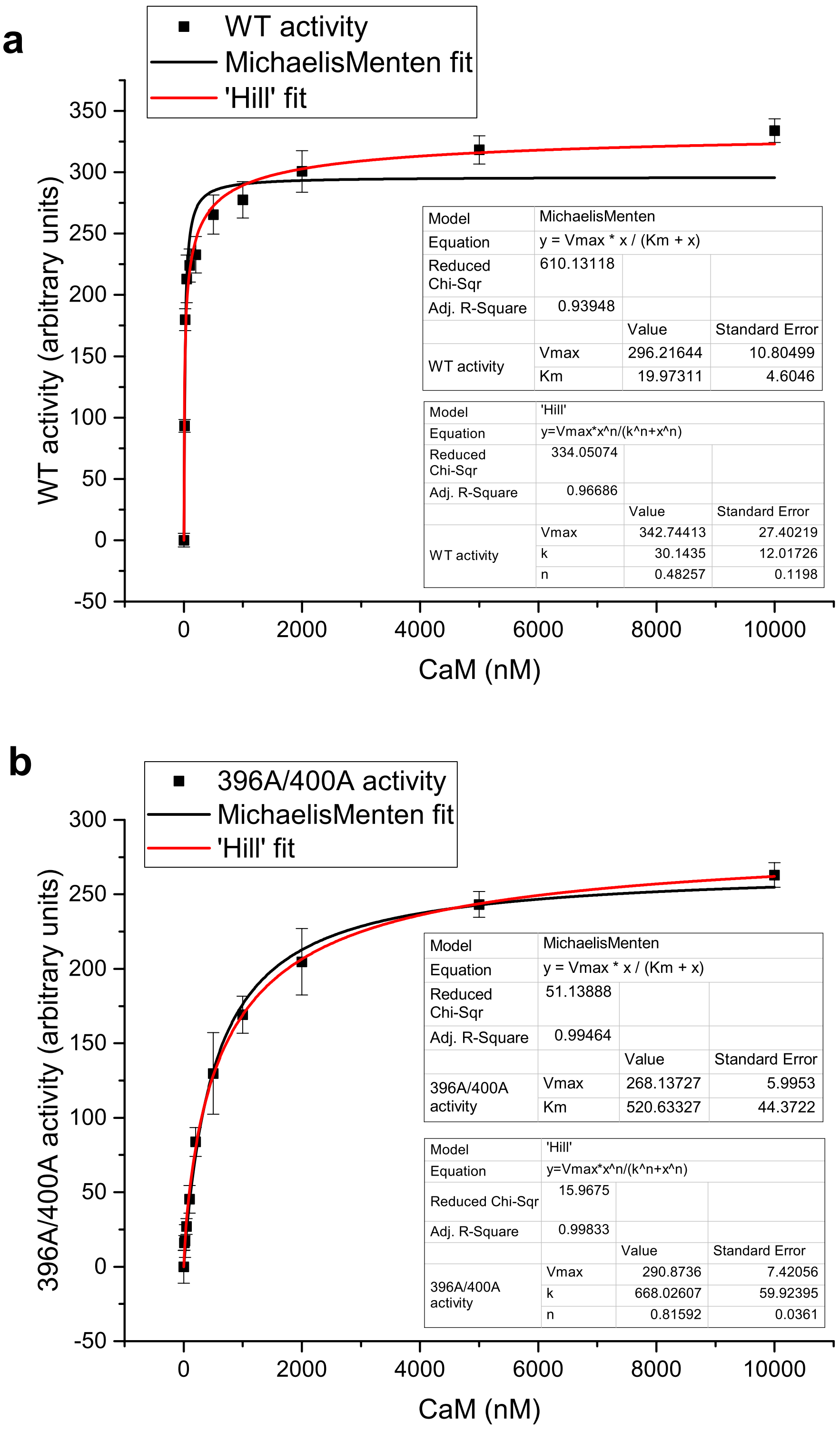

Supplementary Figure 3. Relationship of calcineurin WT and I396A/I400A activity to CaM concentration. Detailed results are shown for curve fitting to model the relationship between CaM concentration and either WT (**a**) or I396A/400A (**b**) calcineurin activity (n=3). Curve fitting was performed in ORIGIN using the Michaelis-Menten equation either with (red line) or without (black line) a cooperativity variable ('n'). The cooperativity term substantially improved the fit for WT calcineurin, so K_{CaM} values were derived from these curves (red lines). Calculated parameters are shown in the inset boxes. Error bars show s.e.m.

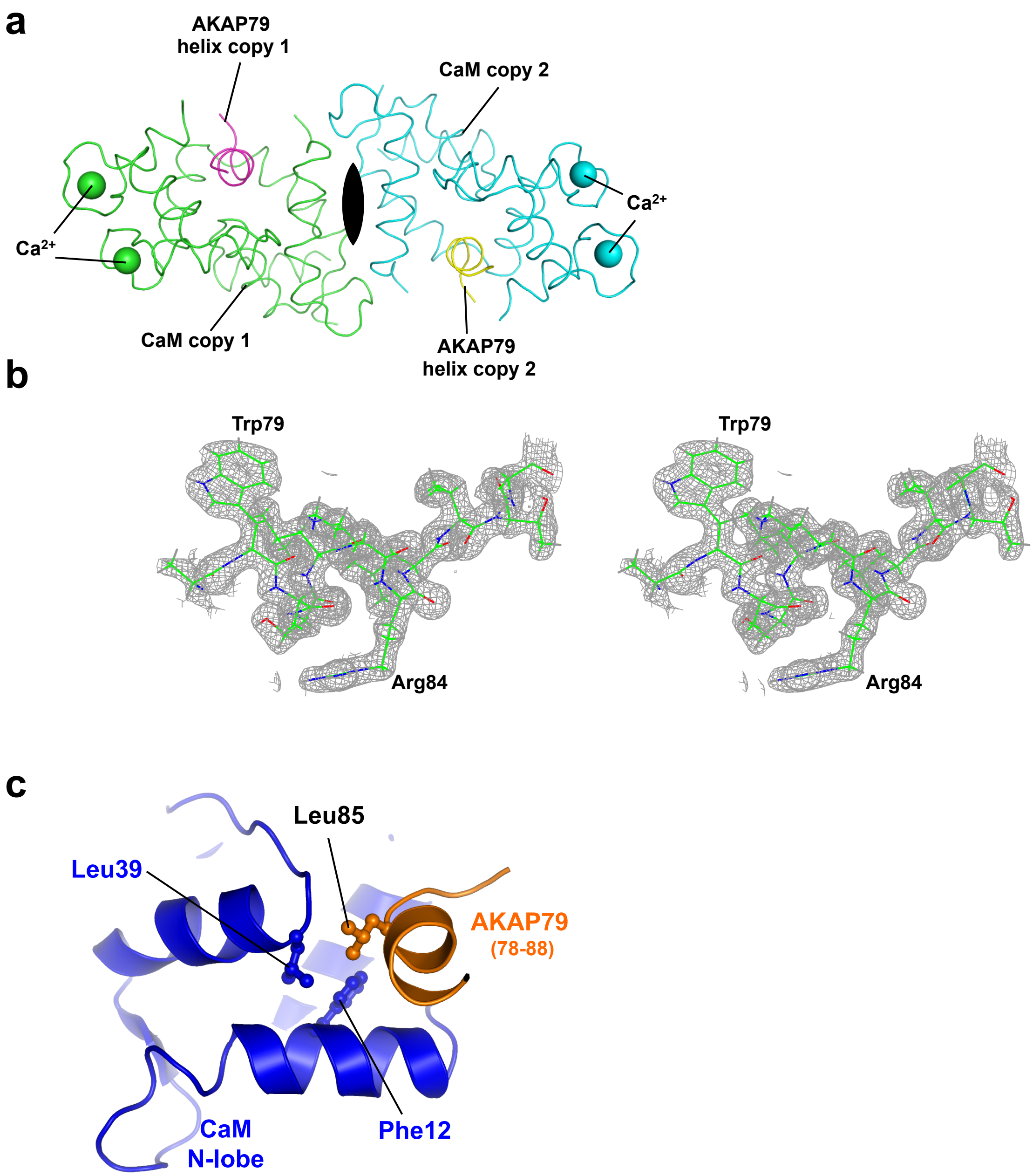

Supplementary Figure 4. Features of the CaM – AKAP79 peptide crystal structure. (**a**) Asymmetric unit showing two copies of the CaM-AKAP79 complex related to each other by a two-fold rotational symmetry axis. Each copy of CaM coordinates two Ca^{2+} ions through its C-lobes as indicated. (**b**) Stereo projections of the $2F_o$-F_c electron density map contoured at 1 σ clipped within 1.5 Å of Chain D (AKAP79 amino acids 78-88) using CCP4MG. (**c**) Close-up highlighting van der Waals interactions between N-lobe amino acids $L39_{CaM}$ and $F12_{CaM}$ and $L85_{AKAP79}$.

P	G	A	C	S	T	V	I	L	M	F	Y	W	H	K	R	Q	N	D	E
1	1	1	1	1	1	1	1	1	1	1	1	1	1	1	1	1	1	1	1
1	1	1	1	1	1	1	1	1	1	1	1	1	1	1	1	1	1	1	1
1	1	1	1	1	1	1	1	1	1	1	1	1	1	1	1	1	1	1	1
1	1	1	1	1	1	1	1	1	1	1	1	1	1	1	1	1	1	1	1
1	1	1	1	1	1	1	1	1	1	1	1	1	1	1	1	1	1	1	1
1	1	1	1	1	1	1	1	1	1	1	1	1	1	1	1	1	1	1	1
1	1	1	1	1	1	1	1	1	1	1	1	1	1	1	1	1	1	1	1
0	0	0	0	0	0	0	0	0	0	0	0	21	0	0	0	0	0	0	0
1	1	1	1	1	1	1	1	1	1	1	1	1	1	1	1	1	1	1	1
0	0	0	0	10	6	0	0	0	0	0	0	0	0	0	0	0	0	5	0
0	0	0	0	0	0	3	3	13	1	1	0	0	0	0	0	0	0	0	0
0.5	0.5	0.5	0.5	0.5	0.5	0.5	0.5	0.5	0.5	0.5	0.5	0.5	0.5	6	6	0.5	0.5	0.5	0.5
0.5	0.5	0.5	0.5	0.5	0.5	0.5	0.5	0.5	0.5	0.5	0.5	0.5	0.5	6	6	0.5	0.5	0.5	0.5
0	0	0	0	0	0	3	3	13	1	1	0	0	0	0	0	0	0	0	0
0	0	0	0	0	0	13	3	3	1	1	0	0	0	0	0	0	0	0	0

Supplementary Figure 5. 1-4-7-8 motif search matrix. The matrix was used as an input to Scansite 3 to search for potential ‘1-4-7-8’-type CaM interaction motifs.

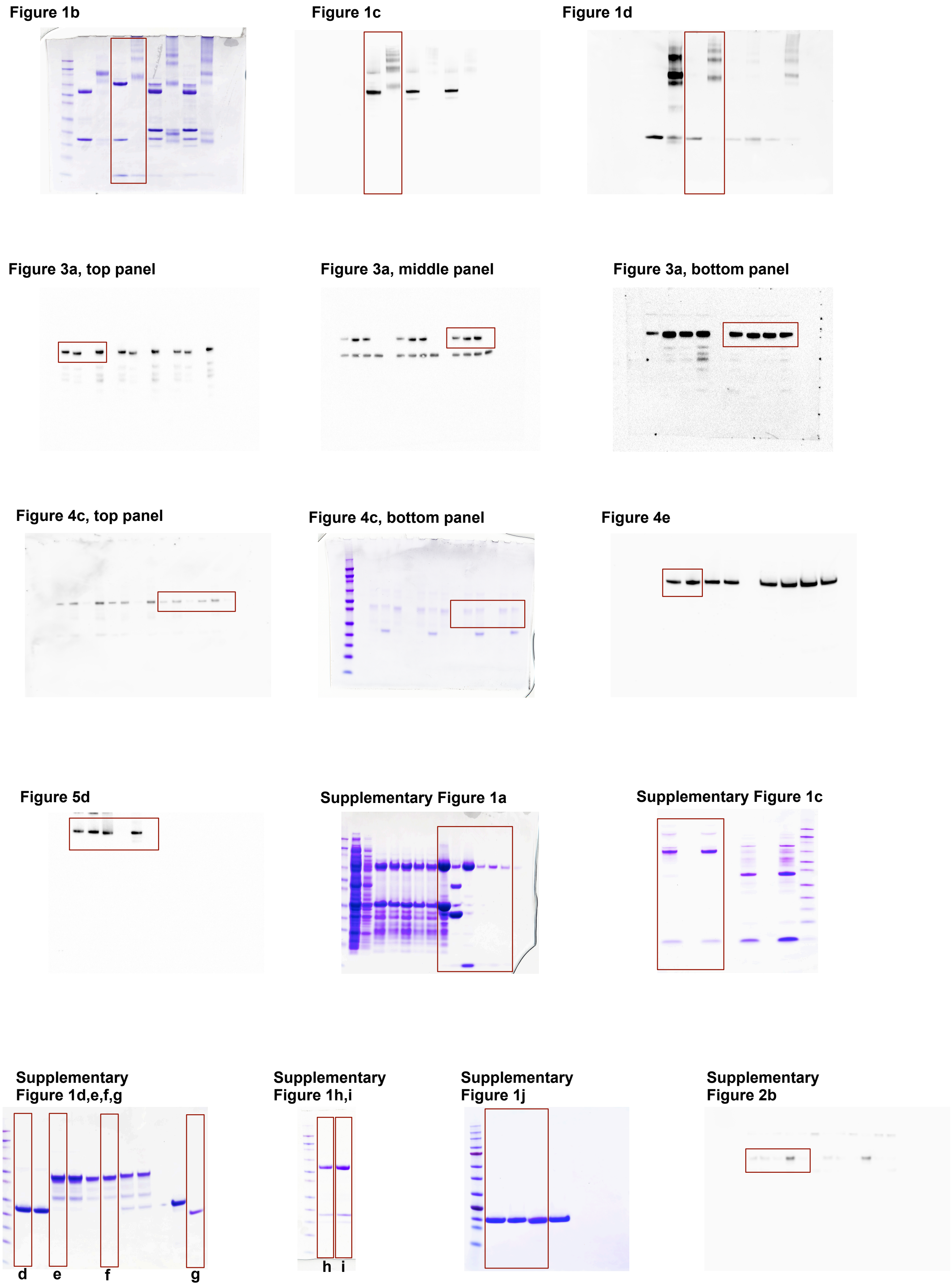

Supplementary Figure 6. Uncropped images of blots and gels used in the main and supplementary figures.

Supplementary Table 1

INTERLINKS

Sequence	Protein 1	Protein 2	Type	Position 1	Position 2	deltaS	Id-Score	False discovery rate (%)	Frequency
KKAAK-VFDKDGNGYISAAELR-a1-b4	AKAP79	Calmodulin	interlink	41	94	0.26	34.49	0	1
EAFSLFDKDGDGTITTK-GAWASLKR-EAFSLFDKDGDGTITTK-a7-b8	AKAP79	Calmodulin	interlink	83	21	0.39	39.2	0	1
KRSESSK-EAFSLFDKDGDGTITTK-a1-b8	AKAP79	Calmodulin	interlink	90	21	0	29.86	0	1
SESSKQQKPLEGEMQPAINAEDADLSK-KMKDTDSEEEIREAFR-a5-b1	AKAP79	Calmodulin	interlink	96	75	0.93	24.69	0	1
SESSKQQKPLEGEMQPAINAEDADLSK-EAFSLFDKDGDGTITTK-a8-b8	AKAP79	Calmodulin	interlink	99	21	0	35.03	0	1
KAKSR-MKDTDSEEEIREAFR-a1-b2	AKAP79	Calmodulin	interlink	120	77	0	36.83	0	1
KAKSR-VFDKDGNGYISAAEL-a1-b4	AKAP79	Calmodulin	interlink	120	94	0.38	29.06	0	1
KAKSR-MKDTDSEEEIREAFR-a3-b2	AKAP79	Calmodulin	interlink	122	77	0	29.69	0	1
VQEEAEILDIQTQTPLNDQATKAK-VFDKDGNGYISAAELR-a22-b4	AKAP79	Calmodulin	interlink	175	94	0.59	31.72	0	1
ALKPKAGSEAADVAR-VFDKDGNGYISAAELR-a3-b4	AKAP79	Calmodulin	interlink	48	94	0.26	36.6	0	2
ALKPK-EAFSLFDKDGDGTITTK-a3-b8	AKAP79	Calmodulin	interlink	48	21	0.22	36.57	0	2
ALKPK-VFDKDGNGYISAAELR-a3-b4	AKAP79	Calmodulin	interlink	48	94	0.26	33.89	0	2
ALKPKAGSEAADVAR-EAFSLFDKDGDGTITTK-a3-b8	AKAP79	Calmodulin	interlink	48	21	0.21	29.63	0	2
ALKPKAGSEAADVAR-VFDKDGNGYISAAELR-a5-b4	AKAP79	Calmodulin	interlink	50	94	0	40.93	0	2
ALKPKAGSEAADVAR-VFDKDGNGYISAAELR-a5-b4	AKAP79	Calmodulin	interlink	50	94	0.12	37.78	0	2
QQKPLEGEMQPAINAEDADLSKK-VFDKDGNGYISAAELR-a22-b4	AKAP79	Calmodulin	interlink	118	94	0.61	32.69	0	2
QQKPLEGEMQPAINAEDADLSKK-VFDKDGNGYISAAELR-a22-b4	AKAP79	Calmodulin	interlink	118	94	0.63	23.54	0	2
AKSTQDLSEGISQK-VFDKDGNGYISAAELR-a2-b4	AKAP79	Calmodulin	interlink	177	94	0.54	41.27	0	2
AKSTQDLSEGISQK-VFDKDGNGYISAAELR-a2-b4	AKAP79	Calmodulin	interlink	177	94	0	37.82	0	2
KRSESSK-VFDKDGNGYISAAELR-a1-b4	AKAP79	Calmodulin	interlink	90	94	0	36.37	0	4
KRSESSKQQKPLEGEMQPAINAEDADLSK-VFDKDGNGYISAAELR-a1-b4	AKAP79	Calmodulin	interlink	90	94	0.75	34.46	0	4
KRSESSK-VFDKDGNGYISAAELR-a1-b4	AKAP79	Calmodulin	interlink	90	94	0	34.02	0	4
KRSESSK-VFDKDGNGYISAAELR-a1-b4	AKAP79	Calmodulin	interlink	90	94	0	32.45	0	4
SESSKQQKPLEGEMQPAINAEDADLSK-VFDKDGNGYISAAELR-a5-b4	AKAP79	Calmodulin	interlink	96	94	0.77	31.7	0	7
RSESSKQQKPLEGEMQPAINAEDADLSK-VFDKDGNGYISAAELR-a6-b4	AKAP79	Calmodulin	interlink	96	94	0.22	30.77	0	7
RSESSKQQKPLEGEMQPAINAEDADLSK-VFDKDGNGYISAAELR-a6-b4	AKAP79	Calmodulin	interlink	96	94	0.57	28.89	0	7
SESSKQQKPLEGEMQPAINAEDADLSK-VFDKDGNGYISAAELR-a5-b4	AKAP79	Calmodulin	interlink	96	94	0.21	28.36	0	7
SESSKQQKPLEGEMQPAINAEDADLSK-VFDKDGNGYISAAELR-a5-b4	AKAP79	Calmodulin	interlink	96	94	0	28.87	0	7
RSESSKQQKPLEGEMQPAINAEDADLSK-VFDKDGNGYISAAELR-a6-b4	AKAP79	Calmodulin	interlink	96	94	0	27.45	0	7
KRSESSKQQKPLEGEMQPAINAEDADLSK-VFDKDGNGYISAAELR-a7-b4	AKAP79	Calmodulin	interlink	96	94	0.66	26.96	0	7
SESSKQQKPLEGEMQPAINAEDADLSK-VFDKDGNGYISAAELR-a8-b4	AKAP79	Calmodulin	interlink	99	94	0.7	39.34	0	16
SESSKQQKPLEGEMQPAINAEDADLSKK-VFDKDGNGYISAAELR-a8-b4	AKAP79	Calmodulin	interlink	99	94	0.49	35.29	0	16
QQKPLEGEMQPAINAEDADLSK-VFDKDGNGYISAAELR-a3-b4	AKAP79	Calmodulin	interlink	99	94	0.59	32.22	0	16
SESSKQQKPLEGEMQPAINAEDADLSK-VFDKDGNGYISAAELR-a8-b4	AKAP79	Calmodulin	interlink	99	94	0.71	40.16	0	16
RSESSKQQKPLEGEMQPAINAEDADLSK-VFDKDGNGYISAAELR-a9-b4	AKAP79	Calmodulin	interlink	99	94	0	36.95	0	16
SESSKQQKPLEGEMQPAINAEDADLSK-VFDKDGNGYISAAELR-a8-b4	AKAP79	Calmodulin	interlink	99	94	0.68	36.18	0	16
QQKPLEGEMQPAINAEDADLSK-VFDKDGNGYISAAELR-a3-b4	AKAP79	Calmodulin	interlink	99	94	0	29.09	0	16
KRSESSKQQKPLEGEMQPAINAEDADLSK-VFDKDGNGYISAAELR-a10-b4	AKAP79	Calmodulin	interlink	99	94	0.64	26.25	0	16
SESSKQQKPLEGEMQPAINAEDADLSKK-VFDKDGNGYISAAELR-a8-b4	AKAP79	Calmodulin	interlink	99	94	0.48	51.49	0	16
SESSKQQKPLEGEMQPAINAEDADLSK-VFDKDGNGYISAAELR-a8-b4	AKAP79	Calmodulin	interlink	99	94	0.73	34.2	0	16
QQKPLEGEMQPAINAEDADLSKK-VFDKDGNGYISAAELR-a3-b4	AKAP79	Calmodulin	interlink	99	94	0.58	32.98	0	16
RSESSKQQKPLEGEMQPAINAEDADLSK-VFDKDGNGYISAAELR-a9-b4	AKAP79	Calmodulin	interlink	99	94	0	32.18	0	16
KRSESSKQQKPLEGEMQPAINAEDADLSK-VFDKDGNGYISAAELR-a10-b4	AKAP79	Calmodulin	interlink	99	94	0.52	30.04	0	16
QQKPLEGEMQPAINAEDADLSK-VFDKDGNGYISAAELR-a3-b4	AKAP79	Calmodulin	interlink	99	94	0	29.99	0	16
SESSKQQKPLEGEMQPAINAEDADLSK-EAFSLFDKDGDGTITTK-a5-b8	AKAP79	Calmodulin	interlink	96	21	0	27.14	0.042	1
QQKPLEGEMQPAINAEDADLSKK-VFDKDGNGYISAAELR-a3-b4	AKAP79	Calmodulin	interlink	99	94	0.62	26.53	0.042	16
RSESSKQQKPLEGEMQPAINAEDADLSK-VFDKDGNGYISAAELR-a9-b4	AKAP79	Calmodulin	interlink	99	94	0.53	25.01	0.042	16

INTRALINKS

Sequence	Protein 1	Protein 2	Type	Position 1	Position 2	deltaS	Id-Score	False discovery rate (%)
ALKPKAGSEAADVAR-AKSTQDLSEGISQK-a5-b2	AKAP79	AKAP79	intralink	50	177	0.42	44.06	0
ALKPKAGSEAADVAR-KKAAK-a5-b2	AKAP79	AKAP79	intralink	50	42	0.42	43.4	0
ALKPKAGSEAADVAR-ALKPK-a5-b3	AKAP79	AKAP79	intralink	50	48	0.37	43.08	0
SKSEESK-SKNVPK-a2-b2	AKAP79	AKAP79	intralink	328	356	0.68	41.85	0
ALKPKAGSEAADVAR-AAKALKPK-a5-b3	AKAP79	AKAP79	intralink	50	45	0.8	41.29	0
ALKPKAGSEAADVAR-GAWASLKR-a5-b7	AKAP79	AKAP79	intralink	50	83	0.55	40.83	0
SKSEESKR-SKNVPK-a2-b2	AKAP79	AKAP79	intralink	328	356	0	39.63	0
AAKALKPKAGSEAADVAR-ALKPK-a8-b3	AKAP79	AKAP79	intralink	50	48	0.94	39.23	0
SKSEESKR-SKNVPK-a7-b2	AKAP79	AKAP79	intralink	333	356	0	38.39	0
ALKPKAGSEAADVAR-KRSESSK-a5-b1	AKAP79	AKAP79	intralink	50	90	0	38.08	0
AAKALKPK-SKNVPK-a3-b2	AKAP79	AKAP79	intralink	45	356	0	37.78	0
AKSTQDLSEGISQK-ALKPK-a2-b3	AKAP79	AKAP79	intralink	177	48	0.18	37.73	0
AAKALKPK-ALKPK-a3-b3	AKAP79	AKAP79	intralink	45	48	0	37.69	0
AKSTQDLSEGISQK-KKAAK-a2-b2	AKAP79	AKAP79	intralink	177	42	0.21	37.5	0
AKSTQDLSEGISQK-KKAAK-a2-b1	AKAP79	AKAP79	intralink	177	41	0.42	37.46	0
ALKPKAGSEAADVAR-KKAAK-a5-b1	AKAP79	AKAP79	intralink	50	41	0.63	37.34	0
ALKPKAGSEAADVAR-SKSEESKR-a5-b7	AKAP79	AKAP79	intralink	50	333	0	37.26	0
SESSKQQKPLEGEMQPAINAEDADLSK-GAWASLKR-a5-b7	AKAP79	AKAP79	intralink	96	83	0.81	37.19	0
ALKPKAGSEAADVAR-SKNVPK-a5-b2	AKAP79	AKAP79	intralink	50	356	0.31	37.14	0
AKSTQDLSEGISQK-KAKSR-a2-b3	AKAP79	AKAP79	intralink	177	122	0.36	36.76	0
AAKALKPK-GAWASLKR-a3-b7	AKAP79	AKAP79	intralink	45	83	0.53	36.58	0
GAWASLKR-ALKPK-a7-b3	AKAP79	AKAP79	intralink	83	48	0	36.54	0
GAWASLKR-GAWASLKR-a7-b7	AKAP79	AKAP79	intralink	83	83	0.31	36.02	0
MEPIAIIITDTEISEFDVTKSK-SKSEESKR-a20-b7	AKAP79	AKAP79	intralink	354	333	0.51	35.75	0
SESSKQQKPLEGEMQPAINAEDADLSKK-GAWASLKR-a5-b7	AKAP79	AKAP79	intralink	96	83	0	35.63	0
GAWASLKR-KKAAK-a7-b2	AKAP79	AKAP79	intralink	83	42	0	35.6	0
SEESKR-SKNVPK-a5-b2	AKAP79	AKAP79	intralink	333	356	0	35.3	0
QQKPLEGEMQPAINAEDADLSK-KRSESSK-a3-b1	AKAP79	AKAP79	intralink	99	90	0.21	34.91	0
GAWASLKR-KRSESSK-a7-b1	AKAP79	AKAP79	intralink	83	90	0	34.65	0
SESSKQQKPLEGEMQPAINAEDADLSK-AKSTQDLSEGISQK-a8-b2	AKAP79	AKAP79	intralink	99	177	0.7	34.58	0
QQKPLEGEMQPAINAEDADLSKK-KAKSR-a22-b3	AKAP79	AKAP79	intralink	118	122	0.25	34.5	0
QQKPLEGEMQPAINAEDADLSKK-KAKSR-a22-b1	AKAP79	AKAP79	intralink	118	120	0.27	34.4	0
DTELSQESDFKENGITEEK-SKSEESK-a11-b2	AKAP79	AKAP79	intralink	318	328	0	33.96	0
ALKPKAGSEAADVAR-KKAAK-a5-b2	AKAP79	AKAP79	intralink	50	44	0.38	44.2	0
ALKPKAGSEAADVAR-GAWASLKR-a5-b7	AKAP79	AKAP79	intralink	50	85	0.57	42.75	0
ALKPKAGSEAADVAR-GAWASLKR-a3-b7	AKAP79	AKAP79	intralink	48	85	0	42.38	0
ALKPKAGSEAADVAR-AKSTQDLSEGISQK-a5-b2	AKAP79	AKAP79	intralink	50	179	0.46	42.27	0
ALKPKAGSEAADVAR-ALKPK-a5-b3	AKAP79	AKAP79	intralink	50	50	0	41.06	0
DTELSQESDFKENGITEEK-SKSEESKR-a11-b7	AKAP79	AKAP79	intralink	318	335	0.84	38.54	0
DTELSQESDFKENGITEEKSK-SEESKR-a19-b5	AKAP79	AKAP79	intralink	326	335	0	38.29	0
DTELSQESDFKENGITEEKSK-SEESKR-a11-b5	AKAP79	AKAP79	intralink	318	335	0	36.24	0
ALKPKAGSEAADVAR-AKSTQDLSEGISQK-a3-b2	AKAP79	AKAP79	intralink	48	179	0.36	36.08	0
DTELSQESDFKENGITEEK-SEESKR-a11-b5	AKAP79	AKAP79	intralink	318	335	0	34.67	0
ALKPKAGSEAADVAR-ALKPKAGSEAADVAR-a5-b5	AKAP79	AKAP79	intralink	50	52	0	34.1	0
SKNVPKQFLISAENEQVGVFANDNGFEDR-SKSEESKR-a6-b2	AKAP79	AKAP79	intralink	360	330	0	33.87	0
NVPKQFLISAENEQVGVFANDNGFEDR-SKSEESKR-a4-b7	AKAP79	AKAP79	intralink	360	335	0	33.11	0
QQKPLEGEMQPAINAEDADLSK-RKRSESSK-a3-b2	AKAP79	AKAP79	intralink	99	92	0	32.93	0
ALKPKAGSEAADVAR-KAKSR-a5-b1	AKAP79	AKAP79	intralink	50	122	0	32.2	0
SESSKQQKPLEGEMQPAINAEDADLSKK-KAKSR-a27-b3	AKAP79	AKAP79	intralink	118	124	0.56	32.16	0
AKSTQDLSEGISQK-ALKPK-a2-b3	AKAP79	AKAP79	intralink	177	50	0	32.04	0
ALKPKAGSEAADVAR-AAKALKPK-a5-b3	AKAP79	AKAP79	intralink	50	47	0.78	30.98	0
QQKPLEGEMQPAINAEDADLSKK-RKRSESSK-a3-b2	AKAP79	AKAP79	intralink	99	92	0	30.36	0
SESSKQQKPLEGEMQPAINAEDADLSK-ALKPKAGSEAADVAR-a5-b5	AKAP79	AKAP79	intralink	96	52	0.17	29.77	0
SESSKQQKPLEGEMQPAINAEDADLSK-GAWASLKR-a5-b7	AKAP79	AKAP79	intralink	96	85	0	28.8	0
SESSKQQKPLEGEMQPAINAEDADLSK-ALKPKAGSEAADVAR-a5-b3	AKAP79	AKAP79	intralink	96	50	0.19	28.7	0
QQKPLEGEMQPAINAEDADLSK-SKSEESKR-a3-b7	AKAP79	AKAP79	intralink	99	335	0.44	27.47	0
QQKPLEGEMQPAINAEDADLSK-SKSEESKR-a3-b2	AKAP79	AKAP79	intralink	99	330	0.52	26.98	0
NVPKQFLISAENEQVGVFANDNGFEDR-SKSEESKR-a4-b2	AKAP79	AKAP79	intralink	360	330	0	26.92	0
DTELSQESDFKENGITEEK-SKSEESKR-a11-b2	AKAP79	AKAP79	intralink	318	330	0	26.61	0
ALKPKAGSEAADVAR-KRSESSK-a5-b1	AKAP79	AKAP79	intralink	50	92	0.38	26.29	0
QQKPLEGEMQPAINAEDADLSK-QQKPLEGEMQPAINAEDADLSK-a3-b3	AKAP79	AKAP79	intralink	99	101	0	26.18	0
SESSKQQKPLEGEMQPAINAEDADLSK-KKAKSR-a8-b2	AKAP79	AKAP79	intralink	99	122	0	25.46	0
AKSTQDLSEGISQK-KRSESSK-a2-b1	AKAP79	AKAP79	intralink	177	92	0	25.36	0
DYESTEIVAEETKPKDTELSQESDFKENGITEEK-SKSEESKR-a26-b2	AKAP79	AKAP79	intralink	318	330	0	24.6	0
QQKPLEGEMQPAINAEDADLSKK-KRSESSK-a3-b1	AKAP79	AKAP79	intralink	99	92	0.24	23.98	0
DYESTEIVAEETKPKDTELSQESDFKENGITEEK-SKSEESKR-a26-b7	AKAP79	AKAP79	intralink	318	335	0	23.09	0
QQKPLEGEMQPAINAEDADLSKK-KAKSR-a22-b1	AKAP79	AKAP79	intralink	118	122	0.37	22.45	0
DYESTEIVAEETKPKDTELSQESDFKENGITEEK-SKSEESKR-a13-b2	AKAP79	AKAP79	intralink	305	330	0	21.47	0
RSESSKQQKPLEGEMQPAINAEDADLSK-KKAKSR-a6-b1	AKAP79	AKAP79	intralink	96	121	0	19.51	0
QQKPLEGEMQPAINAEDADLSKK-KAKSR-a22-b3	AKAP79	AKAP79	intralink	118	124	0.6	19.32	0
SESSKQQKPLEGEMQPAINAEDADLSK-GAWASLKR-a8-b7	AKAP79	AKAP79	intralink	99	85	0.83	41.33	0
ALKPKAGSEAADVAR-KKAAK-a5-b2	AKAP79	AKAP79	intralink	50	44	0.46	40.96	0
ALKPKAGSEAADVAR-AKSTQDLSEGISQK-a5-b2	AKAP79	AKAP79	intralink	50	179	0.52	40.55	0
ALKPKAGSEAADVAR-GAWASLKR-a3-b7	AKAP79	AKAP79	intralink	48	85	0	39.71	0
SESSKQQKPLEGEMQPAINAEDADLSKK-GAWASLKR-a5-b7	AKAP79	AKAP79	intralink	96	85	0.57	37.61	0
ALKPKAGSEAADVAR-ALKPK-a5-b3	AKAP79	AKAP79	intralink	50	50	0	36.64	0
QQKPLEGEMQPAINAEDADLSK-RKRSESSK-a3-b2	AKAP79	AKAP79	intralink	99	92	0	36.43	0
ALKPKAGSEAADVAR-KKAAK-a5-b1	AKAP79	AKAP79	intralink	50	43	0.32	35.77	0
DTELSQESDFKENGITEEK-SEESKR-a11-b5	AKAP79	AKAP79	intralink	318	335	0	35.77	0
SKNVPKQFLISAENEQVGVFANDNGFEDR-SKSEESKR-a6-b2	AKAP79	AKAP79	intralink	360	330	0	34.36	0
DTELSQESDFKENGITEEKSK-SEESKR-a11-b5	AKAP79	AKAP79	intralink	318	335	0.87	33.75	0
AKSTQDLSEGISQK-ALKPK-a2-b3	AKAP79	AKAP79	intralink	177	50	0.44	33.27	0
SESSKQQKPLEGEMQPAINAEDADLSK-ALKPKAGSEAADVAR-a5-b3	AKAP79	AKAP79	intralink	96	50	0	32.59	0
QQKPLEGEMQPAINAEDADLSKK-RKRSESSK-a3-b2	AKAP79	AKAP79	intralink	99	92	0.25	31.83	0
DTELSQESDFKENGITEEKSK-SEESKR-a19-b5	AKAP79	AKAP79	intralink	326	335	0.78	31.51	0
SESSKQQKPLEGEMQPAINAEDADLSK-ALKPK-a8-b3	AKAP79	AKAP79	intralink	99	50	0.34	31.42	0
ALKPKAGSEAADVAR-RKKAAK-a5-b3	AKAP79	AKAP79	intralink	50	44	0.36	31.1	0
QQKPLEGEMQPAINAEDADLSK-KRSESSK-a3-b1	AKAP79	AKAP79	intralink	99	92	0.91	31.07	0
ALKPKAGSEAADVAR-KAKSR-a3-b1	AKAP79	AKAP79	intralink	48	122	0	30.68	0

SESSKQQKPLEGEMQPAINAEDADLSK-GAWASLKR-a5-b7	AKAP79	AKAP79	intralink	96	85	0	30.32	0
QQKPLEGEMQPAINAEDADLSKK-KAKSR-a22-b3	AKAP79	AKAP79	intralink	118	124	0.36	28.8	0
ALKPKAGSEAADVAR-ALKPKAGSEAADVAR-a5-b5	AKAP79	AKAP79	intralink	50	52	0	28.7	0
ALKPKAGSEAADVAR-KAKSR-a5-b1	AKAP79	AKAP79	intralink	50	122	0	28.55	0
SESSKQQKPLEGEMQPAINAEDADLSKK-KAKSR-a27-b3	AKAP79	AKAP79	intralink	118	124	0.4	28.23	0
ALKPKAGSEAADVAR-KAKSR-a5-b3	AKAP79	AKAP79	intralink	50	124	0	27.56	0
SKNVPKQFLISAENEQVGVFANDNGFEDR-SKSEESKR-a2-b2	AKAP79	AKAP79	intralink	356	328	0.28	33.89	0.014
AKSTQDLSEGISQK-AAKALKPK-a2-b3	AKAP79	AKAP79	intralink	177	45	0	33.66	0.014
ALKPKAGSEAADVARK-KKAAK-a5-b2	AKAP79	AKAP79	intralink	50	42	0.5	33.59	0.014
DTELSQESDFKENGITEEK-SEESKR-a11-b5	AKAP79	AKAP79	intralink	318	333	0	33.24	0.014
SESSKQQKPLEGEMQPAINAEDADLSKK-GAWASLKR-a8-b7	AKAP79	AKAP79	intralink	99	83	0.2	32.79	0.014
QQKPLEGEMQPAINAEDADLSK-ALKPK-a3-b3	AKAP79	AKAP79	intralink	99	48	0.21	32.61	0.014
GAWASLKR-KAKSR-a7-b3	AKAP79	AKAP79	intralink	83	122	0.58	32.54	0.014
ALKPKAGSEAADVAR-ALKPKAGSEAADVAR-a5-b5	AKAP79	AKAP79	intralink	50	50	0	32.33	0.014
DTELSQESDFKENGITEEKSK-SEESKR-a11-b5	AKAP79	AKAP79	intralink	318	333	0.85	32.22	0.014
DTELSQESDFKENGITEEKSK-SEESKR-a19-b5	AKAP79	AKAP79	intralink	326	333	0.23	31.37	0.014
GAWASLKR-SKNVPK-a7-b2	AKAP79	AKAP79	intralink	83	356	0.15	31.13	0.014
SESSKQQKPLEGEMQPAINAEDADLSK-ALKPK-a8-b3	AKAP79	AKAP79	intralink	99	48	0.38	30.94	0.014
SESSKQQKPLEGEMQPAINAEDADLSK-GAWASLKR-a8-b7	AKAP79	AKAP79	intralink	99	83	0	30.92	0.014
SKNVPKQFLISAENEQVGVFANDNGFEDR-SKSEESKR-a6-b2	AKAP79	AKAP79	intralink	360	328	0.16	30.83	0.014
ALKPKAGSEAADVAR-GAWASLKR-a3-b7	AKAP79	AKAP79	intralink	48	83	0	30.68	0.014
ALKPK-KKAAK-a3-b2	AKAP79	AKAP79	intralink	48	42	0	30.63	0.014
VQEEAEILDIQTQTPLNDQATKAK-AAKALKPK-a22-b3	AKAP79	AKAP79	intralink	175	45	0	30.29	0.014
ENGITEEKSK-SEESKR-a8-b5	AKAP79	AKAP79	intralink	326	333	0.48	30.18	0.014
SKSEESKR-ALKPK-a7-b3	AKAP79	AKAP79	intralink	333	48	0	29.99	0.014
ALKPKAGSEAADVAR-KAKSR-a5-b1	AKAP79	AKAP79	intralink	50	120	0.36	29.83	0.014
ALKPKAGSEAADVAR-KAKSR-a5-b3	AKAP79	AKAP79	intralink	50	122	0.19	29.82	0.014
VQEEAEILDIQTQTPLNDQATKAK-ALKPKAGSEAADVAR-a22-b5	AKAP79	AKAP79	intralink	175	50	0.24	29.49	0.014
SESSKQQKPLEGEMQPAINAEDADLSK-ALKPKAGSEAADVAR-a8-b3	AKAP79	AKAP79	intralink	99	48	0	28.32	0.014
DTELSQESDFKENGITEEK-SKSEESKR-a11-b2	AKAP79	AKAP79	intralink	318	328	0	28.21	0.014
ALKPK-ALKPK-a3-b3	AKAP79	AKAP79	intralink	48	48	0	27.43	0.014
SAEGSPGAERQKEK-AAKALKPK-a12-b3	AKAP79	AKAP79	intralink	29	45	0.4	27.18	0.014
SESSKQQKPLEGEMQPAINAEDADLSK-ALKPKAGSEAADVAR-a5-b3	AKAP79	AKAP79	intralink	96	48	0	27.18	0.014
SESSKQQKPLEGEMQPAINAEDADLSKK-ALKPK-a5-b3	AKAP79	AKAP79	intralink	96	48	0	27.05	0.014
SESSKQQKPLEGEMQPAINAEDADLSKK-KAKSR-a27-b1	AKAP79	AKAP79	intralink	118	120	0.22	26.98	0.014
ALKPK-KKAAK-a3-b1	AKAP79	AKAP79	intralink	48	41	0	26.74	0.014
DYESTEIVAEETKPKDTELSQESDFKENGITEEK-SKSEESKR-a26-b7	AKAP79	AKAP79	intralink	318	333	0	26.55	0.014
QQKPLEGEMQPAINAEDADLSKK-KRSESSK-a3-b1	AKAP79	AKAP79	intralink	99	90	0.3	26.45	0.014
ALKPKAGSEAADVAR-SAEGSPGAERQKEK-a5-b12	AKAP79	AKAP79	intralink	50	29	0.55	26.17	0.014
DTELSQESDFKENGITEEK-SKSEESKR-a11-b7	AKAP79	AKAP79	intralink	318	333	0.8	25.42	0.014
SKSEESKR-ALKPK-a2-b3	AKAP79	AKAP79	intralink	328	48	0	24.74	0.014
QQKPLEGEMQPAINAEDADLSKKK-GAWASLKR-a23-b7	AKAP79	AKAP79	intralink	119	83	0	23.91	0.014
SESSKQQKPLEGEMQPAINAEDADLSK-KKAKSR-a8-b4	AKAP79	AKAP79	intralink	99	124	0	18.57	0.021
QQKPLEGEMQPAINAEDADLSK-DYESTEIVAEETKPK-a3-b13	AKAP79	AKAP79	intralink	99	307	0	18.39	0.021
DYESTEIVAEETKPKDTELSQESDFKENGITEEK-SKSEESKR-a26-b2	AKAP79	AKAP79	intralink	318	328	0.8	22.79	0.042
SESSKQQKPLEGEMQPAINAEDADLSK-KKAKSR-a8-b1	AKAP79	AKAP79	intralink	99	121	0	17.64	0.042
GAWASLKR-SKSEESKR-a7-b7	AKAP79	AKAP79	intralink	83	333	0	22.69	0.049
QQKPLEGEMQPAINAEDADLSKK-GAWASLKR-a22-b7	AKAP79	AKAP79	intralink	118	83	0.84	21.52	0.049
QQKPLEGEMQPAINAEDADLSKKK-KRSESSK-a3-b1	AKAP79	AKAP79	intralink	99	90	0	20.94	0.049
DYESTEIVAEETKPKDTELSQESDFKENGITEEK-SKSEESKR-a26-b2	AKAP79	AKAP79	intralink	318	328	0	20.71	0.049
MHHHHHHMETTISEIHVENKDEKR-SAEGSPGAERQKEK-a20-b12	AKAP79	AKAP79	intralink	13	29	0	20.37	0.049
QQKPLEGEMQPAINAEDADLSKKK-GAWASLKR-a22-b7	AKAP79	AKAP79	intralink	118	83	0	20.31	0.049
SESSKQQKPLEGEMQPAINAEDADLSK-DYESTEIVAEETKPK-a8-b13	AKAP79	AKAP79	intralink	99	305	0.22	19.22	0.049
QQKPLEGEMQPAINAEDADLSKK-KRSESSK-a3-b1	AKAP79	AKAP79	intralink	99	92	0	24.46	0.053
QQKPLEGEMQPAINAEDADLSK-GAWASLKR-a3-b7	AKAP79	AKAP79	intralink	99	85	0	24.34	0.053
VQEEAEILDIQTQTPLNDQATKAK-ALKPKAGSEAADVAR-a22-b5	AKAP79	AKAP79	intralink	175	52	0.69	24.22	0.053
QQKPLEGEMQPAINAEDADLSKK-KAKSR-a22-b1	AKAP79	AKAP79	intralink	118	122	0.29	23.48	0.053
VQEEAEILDIQTQTPLNDQATKAK-GAWASLKR-a22-b7	AKAP79	AKAP79	intralink	175	85	0.56	23.46	0.053
DYESTEIVAEETKPKDTELSQESDFKENGITEEK-SKSEESK-a26-b2	AKAP79	AKAP79	intralink	318	330	0.93	22.36	0.053
ALKPKAGSEAADVAR-KRSESSK-a5-b1	AKAP79	AKAP79	intralink	50	92	0	21.61	0.053
DYESTEIVAEETKPKDTELSQESDFKENGITEEK-SKSEESKR-a26-b7	AKAP79	AKAP79	intralink	318	335	0	21.25	0.053
DTELSQESDFKENGITEEK-SKSEESKR-a11-b2	AKAP79	AKAP79	intralink	318	330	0.89	21.18	0.053
SESSKQQKPLEGEMQPAINAEDADLSK-KKAKSR-a8-b4	AKAP79	AKAP79	intralink	99	124	0	21	0.053
DYESTEIVAEETKPKDTELSQESDFKENGITEEK-SKSEESKR-a26-b2	AKAP79	AKAP79	intralink	318	330	0.75	20.31	0.053
HVMTNLGEKLTDEEVDEMIR-VFDKDGNGYISAAELR-a9-b4	Calmodulin	Calmodulin	intralink	115	94	0.67	43.41	0
KMKDTDSEEEIREAFR-VFDKDGNGYISAAELR-a3-b4	Calmodulin	Calmodulin	intralink	77	94	0.94	41.32	0
KMKDTDSEEEIREAFR-VFDKDGNGYISAAELR-a1-b4	Calmodulin	Calmodulin	intralink	75	94	0.89	39.07	0
EAFRVFDKDGNGYISAAELR-MKDTDSEEEIR-a8-b2	Calmodulin	Calmodulin	intralink	94	77	0.48	35.47	0
EAFRVFDKDGNGYISAAELR-KMKDTDSEEEIR-a8-b3	Calmodulin	Calmodulin	intralink	94	77	0.94	33.77	0
EAFRVFDKDGNGYISAAELR-KMKDTDSEEEIR-a8-b1	Calmodulin	Calmodulin	intralink	94	75	0.94	31.6	0
HVMTNLGEKLTDEEVDEMIR-KMKDTDSEEEIREAFR-a9-b3	Calmodulin	Calmodulin	intralink	115	77	0	26.47	0
VFDKDGNGYISAAELR-MKDTDSEEEIREAFR-a4-b2	Calmodulin	Calmodulin	intralink	94	77	0	21.71	0
KMKDTDSEEEIREAFR-VFDKDGNGYISAAELR-a3-b4	Calmodulin	Calmodulin	intralink	77	94	0.78	38.38	0
KMKDTDSEEEIREAFR-VFDKDGNGYISAAELR-a1-b4	Calmodulin	Calmodulin	intralink	75	94	0.88	37.86	0
EAFRVFDKDGNGYISAAELR-MKDTDSEEEIR-a8-b2	Calmodulin	Calmodulin	intralink	94	75	0	32.87	0.014
EAFRVFDKDGNGYISAAELR-KMKDTDSEEEIR-a8-b3	Calmodulin	Calmodulin	intralink	94	77	0.87	32.7	0.014
VFDKDGNGYISAAELR-MKDTDSEEEIREAFR-a4-b2	Calmodulin	Calmodulin	intralink	94	77	0.9	20.2	0.049

Supplementary Table 1. AKAP79-CaM crosslinks identified by XL-MS. Interlinks and intralinks detected in the crosslinked AKAP79-D/D-CaM sample using XL-MS are listed. Crosslinked peptides were pooled from runs of three biological replicates. deltaS is a measure for how close the best assigned sequence match was scored in regard to the second match. The Id-score is a weighted sum of different measures for assessing the quality of the composite MS2 spectrum calculated by xQuest. FDR denotes the false-discovery rate as calculated by xProphet. Frequency indicates the total number of occasions that a unique crosslink was independently detected within biological replicates.

Supplementary Table 2

Rank	PDB ID/Chain	Z Score	R.M.S. Deviation (Å)	Length of Alignment	Length of aligned protein	Identity (%)	Aligned Protein (region)	Metal Coordination State	Comments
Search performed with N-lobe (2-79) of Chain B from CaM - AKAP79 (77-92) structure									
1	3gn4-D	13.9	1	72	145	100	Calmodulin (N-lobe)	Ca^{2+}-free conformation with all four EF hands coordinating Mg^{2+}	Myosin-VI complex
2	2pq3-A	13.6	0.9	69	74	100	Calmodulin (N-lobe)	Zinc-trapped intermediate	Crystal contains CaM N-terminus only
3	4oy4-A	13.2	0.9	72	334	100	Calmodulin (N-lobe)	EF hands unoccupied	CaM fused within sequence of CaMPARI v0.2 genetically-encoded sensor
4	1cfd-A	13.1	1.1	72	148	100	Calmodulin (N-lobe)	EF hands unoccupied	Solution structure
5	1f70-A	13.1	0.8	69	76	100	Calmodulin (N-lobe)	EF hands unoccupied	N-terminal domain only, solution structure
6	1top-A	12.9	1.1	72	162	68	Troponin C (N-lobe)	N-lobe EF hands are unoccupied, both C-lobe EF hands coordinate Ca^{2+}	Chicken skeletal muscle troponin C
7	2ggm-A	12.9	1.1	72	149	46	Centrin-2 (N-lobe)	N-lobe EF hands are unoccupied, both C-lobe EF hands coordinate Ca^{2+}	Complex with xeroderma pigmentosum group C protein, only contacts to peptide involve C-lobe
8	1lkj-A	12.6	1.4	71	146	62	Calmodulin (N-lobe)	EF hands unoccupied	Yeast CaM (solution structure)
9	2lmv-A	12.5	1.7	72	147	65	Calmodulin-related protein 97A (N-lobe)	N-lobe binds a single Ca^{2+}, both C-lobe EF hands coordinate Ca^{2+}	Androcam binds myosin VI insert 2 site at all Ca^{2+} levels
10	4okh-C	11.9	1.4	71	147	20	Calpain-3 (EF3 and EF4)	EF3 coordinates Ca^{2+}, EF4 is unoccupied	Calpain-3 penta-EF hand domain
11	1xfu-O	11.9	1.5	72	146	100	Calmodulin (N-lobe)	N-lobe closed but binds one Ca^{2+}, both C-lobe EF hands bind Ca^{2+}	Anthrax edema factor (EF) complex
12	1dmo-A	11.8	1.7	71	148	100	Calmodulin (N-lobe)	EF hands unoccupied	Solution structure
13	3fwc-E	11.7	1.7	72	150	36	Cell division control protein 31 (N-lobe)	EF hands unoccupied	Within the Sac3:Sus1:Cdc31 complex
14	2ekw-C	11.2	1.5	69	156	33	Myosin catalytic light chain LC-1 (N-lobe)	EF1 coordinates Ca^{2+}, EF hands 2-4 are unoccupied	Squid myosin S1 complex
15	1juo-B	11.2	1.2	70	172	19	Sorcin (EF3 and EF4)	EF hands unoccupied	Penta-EF-Hand protein
16	1kfu-L	10.9	0.9	70	699	19	M-calpain small subunit (EF3 and EF4)	EF hands unoccupied	m-Calpain Form II complex
17	2bl0-B	10.8	1.1	63	145	38	Myosin regulatory light chain (N-lobe)	EF1 coordinates Ca^{2+}, EF hands 2-4 are unoccupied	Myosin II complex
18	1w7i-B	10.7	2	72	145	44	Myosin LC-1, slow twitch muscle A isoform (N-lobe)	EF hands unoccupied	Myosin V complex
19	1f54-A	10.7	1.4	69	77	62	Calmodulin (N-lobe)	EF hands unoccupied	N-terminus only, solution structure of yeast CaM
20	1br1-F	10.6	1.9	70	148	51	Myosin light polypeptide 6 (N-lobe)	EF hands unoccupied	Myosin XI complex
Search performed with C-lobe (80-147) of Chain B from CaM - AKAP79 (77-92) structure									
1	3sg6-A	14.6	0.5	66	383	100	Calmodulin (C-lobe)	Fully occupied ($4Ca^{2+}$)	CaM fused within sequence of GCaMP2-LIA
2	4q5u-A	14.5	0.5	67	145	100	Calmodulin (C-lobe)	Fully occupied ($4Ca^{2+}$)	CaM bound to its recognition site from calcineurin (1:1 conformation)
3	3u0k-A	14.4	0.6	65	397	100	Calmodulin (C-lobe)	Fully occupied ($4Ca^{2+}$)	CaM fused within sequence of RCaMP
4	1mxe-B	14.4	0.7	67	144	96	Calmodulin (C-lobe)	Fully occupied ($4Ca^{2+}$)	*Drosophila* CaM bound to target sequence of CaM-dependent protein kinase I
5	3oxq-C	14.4	0.7	67	145	100	Calmodulin (C-lobe)	Fully occupied ($4Ca^{2+}$)	Match is to CaM bound to CaV1.2 IQ domain sequence
6	3cln-A	14.4	0.7	67	143	99	Calmodulin (C-lobe)	Fully occupied ($4Ca^{2+}$)	First high-resolution structure of Ca^{2+}-CaM
7	3ek7-A	14.2	0.5	66	389	100	Calmodulin (C-lobe)	Fully occupied ($4Ca^{2+}$)	CaM fused within sequence of GCaMP2
8	2vay-A	14.2	0.7	67	146	100	Calmodulin (C-lobe)	Fully occupied ($4Ca^{2+}$)	CaM bound to Cav1.1 IQ domain
9	3dvk-A	14.2	0.7	67	143	100	Calmodulin (C-lobe)	Fully occupied ($4Ca^{2+}$)	CaM bound to CaV2.3 IQ domain
10	4zlk-B	14.2	0.6	66	133	100	Calmodulin (C-lobe)	Fully occupied ($4Ca^{2+}$)	CaM bound to Myosin VA
11	2be6-C	14.2	0.5	65	136	100	Calmodulin (C-lobe)	Fully occupied ($4Ca^{2+}$)	CaM bound to CaV1.2 IQ domain
12	4aqr-A	14.1	0.6	67	145	87	Calmodulin-7 (C-lobe)	Fully occupied ($4Ca^{2+}$)	In complex with the regulatory domain of a plasma-membrane Ca^{2+}-ATPase
13	3sjq-A	14.1	0.6	67	147	100	Calmodulin (C-lobe)	Fully occupied ($4Ca^{2+}$)	Bound to small conductance potassium channel splice variant (2:2 conformation)
14	3wlc-A	14	0.6	66	392	97	Calmodulin (C-lobe)	Fully occupied ($4Ca^{2+}$)	CaM fused within sequence of GCaMP6m
15	1niw-G	14	0.9	67	145	94	Calmodulin (C-lobe)	Fully occupied ($4Ca^{2+}$)	CaM bound to its recognition site in endothelial nitric oxide synthase
16	2lv6-A	14	0.7	67	148	97	Calmodulin (C-lobe)	Fully occupied ($4Ca^{2+}$)	CaM bound to skeletal muscle myosin light chain kinase
17	4ehq-A	14	0.7	66	145	100	Calmodulin (C-lobe)	Fully occupied ($4Ca^{2+}$)	CaM bound to Ora1 peptide (no interactions with N-lobe)
18	3sui-A	14	1	67	146	100	Calmodulin (C-lobe)	Fully occupied ($4Ca^{2+}$)	CaM bound to trpv1 c-terminal peptide
19	4rjd-A	14	0.7	66	66	100	Calmodulin (C-lobe)	Both EF hands coordinate Ca2+	CaM C-lobe bound to trifluoperazine
20	1exr-A	14	0.7	67	146	84	Calmodulin (C-lobe)	Fully occupied ($4Ca^{2+}$)	Ca^{2+}-bound CaM at atomic (1 Å) resolution
Search performed with full-length (2-147) Chain B from CaM - AKAP79 (77-92) structure									
1	1mxe-B	14.7	2.6	103	144	75	Calmodulin	Fully occupied ($4Ca^{2+}$)	*Drosophila* CaM bound to target sequence of CaM-dependent protein kinase I
2	3sg5-A	14.6	3.6	129	387	100	Calmodulin	Fully occupied ($4Ca^{2+}$)	CaM fused within sequence of GCaMP3 variant
3	4q5u-A	14.5	3.3	135	145	96	Calmodulin	Fully occupied ($4Ca^{2+}$)	CaM bound to its recognition site from calcineurin (1:1 conformation)
4	2w73-A	14.5	12.1	93	145	88	Calmodulin (C-lobes aligned)	Fully occupied ($4Ca^{2+}$)	CaM bound to its recognition site from calcineurin (2:2 conformation)
5	3oxq-C	14.4	3.1	136	145	100	Calmodulin	Fully occupied ($4Ca^{2+}$)	Match is to CaM bound to CaV1.2 IQ domain sequence
6	3sg6-A	14.4	3.6	129	383	100	Calmodulin	Fully occupied ($4Ca^{2+}$)	CaM fused within sequence of GCaMP2-LIA
7	4aqr-A	14.4	4.3	129	145	88	Calmodulin-7	Fully occupied ($4Ca^{2+}$)	In complex with the regulatory domain of a plasma-membrane Ca^{2+}-ATPase
8	3sjq-B	14.4	13.8	122	145	100	Calmodulin	Fully occupied ($4Ca^{2+}$)	Bound to small conductance potassium channel splice variant (2:2 conformation)
9	1ggz-A	14.3	7	90	144	66	Calmodulin-related protein NB-1 (C-lobes aligned)	Fully occupied ($4Ca^{2+}$)	Protein expressed in epithelial cells
10	1osa-A	14.3	14.3	123	148	87	Calmodulin	Fully occupied ($4Ca^{2+}$)	Paramecium CaM
11	3u0k-A	14.2	3.8	131	397	95	Calmodulin	Fully occupied ($4Ca^{2+}$)	CaM fused within sequence of RCaMP
12	5e1k-A	14.2	14.1	123	145	81	Calmodulin	Fully occupied ($4Ca^{2+}$)	Selenomethionine Calmodulin from Paramecium
13	3cln-A	14.2	14.3	120	143	99	Calmodulin	Fully occupied ($4Ca^{2+}$)	First high-resolution structure of Ca^{2+}-CaM
14	1niw-C	14.1	3.2	120	145	80	Calmodulin	Fully occupied ($4Ca^{2+}$)	CaM bound to its recognition site in endothelial nitric oxide synthase
15	2vay-A	14.1	3.3	134	146	100	Calmodulin	Fully occupied ($4Ca^{2+}$)	CaM bound to Cav1.1 IQ domain
16	3dvm-A	14.1	4.8	123	140	89	Calmodulin	Fully occupied ($4Ca^{2+}$)	CaM bound to CaV2.1 IQ domain
17	2obh-B	14.1	11.9	98	143	44	Centrin-2 (C-lobes aligned)	N-lobe EF hands are unoccupied, both C-lobe EF hands coordinate Ca^{2+}	CaM bound to its recognition site from xeroderma pigmentosum group C protein
18	3dvk-A	14	4.7	125	143	87	Calmodulin	Fully occupied ($4Ca^{2+}$)	CaM bound to CaV2.3 IQ domain
19	4zlk-B	14	5.8	115	133	100	Calmodulin	Fully occupied ($4Ca^{2+}$)	CaM bound to Myosin VA
20	3qrx-A	13.9	1.5	70	145	49	Centrin (CaM C-lobe aligned to centrin N-lobe)	Fully occupied ($4Ca^{2+}$)	Bound to mellitin

Supplementary Table 2. Structural homologs identified using DALI. Homologous structures are listed for full-length CaM (2-147), and for isolated N-lobe (2-79) or C-lobe (80-147). Searches were performed using chain B of the crystal structure (5NIN). In each category, the top twenty highest scoring structures are listed. In cases where multiple chains within the same asymmetric unit or NMR assembly scored highly, only the highest scoring chain is listed. For matches to CaM in complex with target proteins/peptides, only the highest scoring PDB match for the complex type is listed (with the exception of 4Q5U and 2W73, which represent respective 1:1 and 2:2 configurations with calcineurin). For matches against non-CaM proteins, e.g. Troponin-C, only the highest scoring PDB match is listed. Structural models deposited in the PDB were excluded from the list.

Supplementary Table 3

Score	Accession	Protein Name	Position of central Trp	Sequence of predicted site	Molecular Weight
0	AKAP5_HUMAN	AKAP79	W79	PEPTRGAwASLKRLVTRRKRSESSKQQKPLEGEMQPA	47094.4
0.183	HNRPM_HUMAN	Heterogeneous nuclear ribonucleoprotein M	W84	NIPFDVKwQSLKDLVKEKVGEVTYVELLMDAEGKSRG	77525.9
0.183	CAC1A_HUMAN	Voltage-dependent P/Q-type calcium channel subunit alpha-1A (Cav2.1)	W597	IFKVTKYwASLRNLVVSLLNSMKSIISLLFLLFLFIV	282400.4
0.183	CAC1B_HUMAN	Voltage-dependent N-type calcium channel subunit alpha-1B (Cav2.2)	W592	IFKVTKYwSSLRNLVVSLLNSMKSIISLLFLLFLFIV	262529.6
0.183	CAC1E_HUMAN	Voltage-dependent R-type calcium channel subunit alpha-1E (Cav2.3)	W586	IFKITKYwASLRNLVVSLMSSMKSIISLLFLLFLFIV	261764.5
0.183	YK026_HUMAN	Putative uncharacterized protein LOC100129027	W112	TPFLTKTwHSLRALVYRVWSLEESRYLQREKGLVDSF	16805.6
0.189	AKA12_HUMAN	A-kinase anchor protein 12 (Gravin)	W759	EGEGVSTwESFKRLVTPRKKSKSKLEEKSEDSIAGSG	191507.2
0.234	PDE1A_HUMAN	Calcium/calmodulin-dependent 3',5'-cyclic nucleotide phosphodiesterase 1A (PDE1A)	W316	INLSKDDwRDLRNLVIEMVLSTDMSGHFQQIKNIRNS	61259.6
0.291	FGL1_HUMAN	Fibrinogen-like protein 1	W292	WYTWHGWwYSLKSVVMKIRPNDFIPNVI (C-term)	36383.9
0.291	BCL2_HUMAN	Apoptosis regulator Bcl-2	W214	RPLFDFSwLSLKTLLSLALVGACITLGAYLGHK (C-term)	26269.3
0.291	DYH10_HUMAN	Dynein heavy chain 10	W4077	QRDPRIPwGSLKYLIGEVMYGGRAIDSFDRRILTIYM	514904.5
0.297	LRFN2_HUMAN	Leucine-rich repeat and fibronectin type-III domain-containing protein 2/Synaptic adhesion-like molecule 1	W166	NNLHGLPwDSVRRMVNLHQLSLDHNLLDHIAEGTFAD	84741.9
0.329	HUS1B_HUMAN	Checkpoint protein HUS1B	W162	ASIRLPRwRTLRSIVERMANVGSHVLVEANLSGRMTL	30985.9
0.329	K1841_HUMAN	Uncharacterized protein KIAA1841	W36	GIPQTINwETIARLVPGLTPKECAKRFDELKSSGSSP	82017.4
0.329	LPAR1_HUMAN	Lysophosphatidic acid receptor 1	W45	GKHLATEwNTVSKLVMGLGITVCIFIMLANLLVMVAI	41114.6
0.329	SCNNB_HUMAN	Amiloride-sensitive sodium channel subunit beta/Epithelial Na(+) channel subunit beta	W549	EIIIDFVwITIIKLVALAKSLRQRRAQASYAGPPPTV	72668.3
0.329	DDX60_HUMAN	Probable ATP-dependent RNA helicase DEAD box protein 60	W412	MKDYEYLwNTVSKLVRDFEVGQPFPLRTTKVCFLEKK	197877.5
0.329	TLR8_HUMAN	Toll-like receptor 8	W1015	PKAEGLFwQTLRNVVLTENDSRYNNMYVDSIKQY (C-term)	119843.1
0.342	KIF1B_HUMAN	Kinesin-like protein KIF1B	W1308	EKGSELHwKDVRELVVGRIRNKPEVDEAAVDAILSLN	204502.2
0.342	BIG2_HUMAN	Brefeldin A-inhibited guanine nucleotide-exchange protein 2 (BIG2)	W1371	HTFEKHWwQDLFRIVFRIFDNMKLPEQLSEKSEWMTT	202063.8
0.342	BIG1_HUMAN	Brefeldin A-inhibited guanine nucleotide-exchange protein 1 (BIG1)	W1424	HTYEKHWwQDLFRIVFRIFDNMKLPEQQTEKAEWMTT	208793.5
0.342	DYHC2_HUMAN	Cytoplasmic dynein 2 heavy chain 1	W1210	EHLSPDHwLDLFRLLGLPRGTSLEKLLFGDLLRVADT	492683.4
0.342	TSNA1_HUMAN	t-SNARE domain-containing protein 1	W188	VVDLKHKwRDLRAVVRRKLGDLRKAAHGPSPGSGKPQ	55956.1
0.342	WDR87_HUMAN	WD repeat-containing protein 87	W10	SPRLIPLwKDLKLLLNDTINKSKQPSEDPKNCLIVLS	333226.1
0.348	ACSM6_HUMAN	Acyl-coenzyme A synthetase ACSM6	W196	SDKSYDGwLDFKKLIQVAPPKQTYMRTKSQDPMAIFF	53591.9
0.366	MFSD7_HUMAN	Major facilitator superfamily domain-containing protein 7	W70	LSMEQINwLSLVYLVVSTPFGVAAIWILDSVGLRAAT	58435.1
0.366	CAC1C_HUMAN	Voltage-dependent L-type calcium channel subunit alpha-1C (Cav1.2)	W634	IFKITRYwNSLSNLVASLLNSVRSIASLLLLLFLFII	249008.3
0.366	CAC1F_HUMAN	Voltage-dependent L-type calcium channel subunit alpha-1F (Cav1.4)	W639	IFKVTRHwASLSNLVASLLNSMKSIASLLLLLFLFII	220706.2
0.366	ANTR1_HUMAN	Anthrax toxin receptor 1	W15	ALGIGFQwLSLATLVLICAGQGGRREDGGPACYGGFD	62797
0.366	BPI_HUMAN	Bactericidal permeability-increasing protein	W15	GPCNAPRwASLMVLVAIGTAVTAAVNPGVVVRISQKG	53906.6
0.366	CAC1D_HUMAN	Voltage-dependent L-type calcium channel subunit alpha-1D (Cav1.3)	W633	IFKVTRHwTSLSNLVASLLNSMKSIASLLLLLFLFII	245171.9
0.366	CAC1S_HUMAN	Voltage-dependent L-type calcium channel subunit alpha-1S (Cav1.1)	W542	IFKITKYwTSLSNLVASLLNSIRSIASLLLLLFLFIV	212376.9
0.366	RTP2_HUMAN	Receptor-transporting protein 2	W205	SLRWCLFwASLCLLVVYLQFSFLSPAFF (C-term)	26070.9
0.372	KCNT2_HUMAN	Potassium channel subfamily T member 2	W156	PFIISIFwPSLRNLFVPVFLNCWLAKHALENMINDLH	130517.4
0.372	ANR17_HUMAN	Ankyrin repeat domain-containing protein 17	W2566	PHAADPSwNSLIKMVSSSTENNGPQTVWTGPWAPHMN	274295.1
0.378	AKA12_HUMAN	A-kinase anchor protein 12 (Gravin)	W610	KREGVTPwASFKKMVTPKKRVRRPSESDKEDELDKVK	191507.2
0.378	THS7B_HUMAN	Thrombospondin type-1 domain-containing protein 7B	W14	LTVTCWVwRSMRKLFLLLSLLLSHAAHLEGKKDNQFI	179425.4
0.399	G137B_HUMAN	Integral membrane protein GPR137B	W88	FLFLCLFwASLRTVLFSFYFKDFVAANSLSPFVFWLL	45604.8
0.399	GP144_HUMAN	Probable G-protein coupled receptor 144	W478	EEQVADTwLSLREVIGGPMALVASVQRLAPLLSTSMT	104101.1
0.399	LCN12_HUMAN	Epididymal-specific lipocalin-12	W10	LLCGLWLwLSLLKVLQAQTPTPLPLPPPMQSFQGNQF	21502.3
0.399	LTN1_HUMAN	E3 ubiquitin-protein ligase listerin	W712	LTKVDLKwNSLLKIIEKACPSSDKHALVTPWLKGDIL	200577.2
0.399	AT12A_HUMAN	Potassium-transporting ATPase alpha chain 2	W433	FDQSSRTwASLSKIITLCNRAEFKPGQENVPIMKKAV	115525.4
0.399	TEX15_HUMAN	Testis-expressed sequence 15 protein	W17	NGDLLLNwTSLKNILSGLNASFPLHNNTGSSTVTTSK	315376.1
0.399	TRIO_HUMAN	Triple functional domain protein	W135	VDMRGSKwDSIKPLLKILQESFPCCIHVALIIKPDNF	346944.3
0.404	KCNC4_HUMAN	Potassium voltage-gated channel subfamily C member 4 (Kv3.4)	W291	IEGVCVLwFTLEFLVRIVCCPDTLDFVKNLLNIIDFV	69775.8
0.404	NYNRI_HUMAN	NYN domain and retroviral integrase catalytic domain-containing protein	W104	LFLDCLCwSTLAYLVPGPPGSLMVGGLTESFIMTQNW	208393.4
0.404	S39A7_HUMAN	Zinc transporter SLC39A7	W18	VAVGLLTwATLGLLVAGLGGHDDLHDDLQEDFHGHSH	50124.8
0.404	S22AF_HUMAN	Solute carrier family 22 member 15	W224	LGYFIRSwRTLAILVNLQGTVVFLLSLFIPESPRWLY	60548
0.404	CBP_HUMAN	CREB-binding protein	W1686	SSLRRSKwSTLCMLVELHTQGQDRFVYTCNECKHHVE	265385.7
0.405	AKA12_HUMAN	A-kinase anchor protein 12 (Gravin)	W804	EPGKEESwVSIKKFIPGRRKKRPDGKQEQAPVEDAGP	191507.2
0.409	DOCK2_HUMAN	Dedicator of cytokinesis protein 2	W380	DSGGQGLwVTMKMLVGDIIQIRKDYPHLVDRTTVVAR	211974.4
0.409	WDR34_HUMAN	WD repeat-containing protein 34	W188	GRLDHGDwSTLKSFVCAWNLDRRDLRPQQPSAVVEVP	57808.2
0.417	FBX44_HUMAN	F-box only protein 44	W37	CRLVCSLwRDLIDLVTLWKRKCLREGFITEDWDQPVA	29750.4
0.417	MPRD_HUMAN	Cation-dependent mannose-6-phosphate receptor	W229	QFPHLAFwQDLGNLVADGCDFVCRSKPRNVPAAYRGV	30997.2
0.417	COCA1_HUMAN	Collagen alpha-1(XII) chain	W138	QKCSVSAwTDLVFLVDGSWSVGRNNFKYILDFIAALV	333190.5
0.423	DYH11_HUMAN	Dynein heavy chain 11	W4139	EANSKVPwEDLRYLFGEIMYGGHITDDWDRKLCRVYL	521107.7
0.423	GRIA4_HUMAN	Glutamate receptor 4 (AMPA 4)	W390	GPRKVGYwNDMDKLVLIQDVPTLGNDTAAIENRTVVV	100883.8
0.423	MDN1_HUMAN	Midasin	W761	TCYRQKRwHDLLRLMQHVHKSAVNKDGKDSETGLLIK	632899.7
0.423	ACSM1_HUMAN	Acyl-coenzyme A synthetase ACSM1, mitochondrial	W196	SDHSREGwLDFRSLVKSASPEHTCVKSKTLDPMVIFF	65281.4
0.423	DYH17_HUMAN	Dynein heavy chain 17, axonemal	W4072	EANPKVPwDDLRYLFGEIMYGGHITDDWDRRLCRTYL	511850.9
0.437	DCD2B_HUMAN	Doublecortin domain-containing protein 2B	W156	SQAASQDwETVLKLLTEKVKLQSGAVCKLCTLEGLPL	37669.9
0.437	MCF2_HUMAN	Proto-oncogene DBL	W186	HRQISGDwQTINKLLTQVHDMETAFDGFWEKHQLKME	107686.4
0.437	ANKY1_HUMAN	Ankyrin repeat and MYND domain-containing protein 1	W527	MIERRKRwRTIKLLLRRGADPNLCCVPMQVLFLAVKA	105529.1
0.437	CALL6_HUMAN	Calmodulin-like protein 6	W129	EGKGYIDwNTLKYVLMNAGEPLNEVEAEQMMKEADKD	20692.8
0.437	TM41A_HUMAN	Transmembrane protein 41A	W219	SLDALFSwDTVFKLLAIAMVALIPGTLIKKFSQKHLQ	29668.9
0.437	SYNE1_HUMAN	Nesprin-1	W5944	LGDLQRSwETLKNVISEKQRTLYEALERQQKYQDSLQ	1011153.6
0.437	UBR1_HUMAN	E3 ubiquitin-protein ligase UBR1	W1558	FLLFQEYwDTVRPLLQRWCADPALLNCLKQKNTVVRY	200235.6
0.442	TAM41_HUMAN	Mitochondrial translocator assembly and maintenance protein 41 homolog	W10	LQTLQSSwVTFRKILSHFPEELSLAFVYGSGVYRQAG	35911.9
0.45	IF172_HUMAN	Intraflagellar transport protein 172 homolog	W1503	CAEAYHSwADLRDVLFNLCENLVKSSEANSPAHEEFK	197600.9
0.45	MYH10_HUMAN	Myosin-10	W619	DRFVAELwKDVDRIVGLDQVTGMTETAFGSAYKTKKG	229027.3
0.45	MYH11_HUMAN	Myosin-11	W619	DKFVADLwKDVDRIVGLDQMAKMTESSLPSASKTKKG	227367.4
0.45	PNML1_HUMAN	PNMA-like protein 1	W121	LDAEGRTwEDVVRLLQLNHPTLSQNQHQPPENWAEAL	48166.9
0.45	BRD1_HUMAN	Bromodomain-containing protein 1	W697	APRRPFSwEDVDRLLDPANRAHLGLEEQLRELLDMLD	119535.2
0.45	TDRD6_HUMAN	Tudor domain-containing protein 6	W580	GNSENVDwYDVRMLLPQFRQLPILAVKCTLADIWPLG	236547.4
0.45	TRI42_HUMAN	Tripartite motif-containing protein 42	W690	NDNGPGQwSDICKVVTPDGHGKNRAKWGLLKNIQSAL	82755.1
0.474	DOP1_HUMAN	Protein dopey-1	W1921	VPNLVDSwASLLILLKDSIQLSLPAPGQFLILGVLNE	277389.8
0.474	ERAP1_HUMAN	Endoplasmic reticulum aminopeptidase 1	W617	VHYEDDGwDSLTGLLKGTHTAVSSNDRASLINNAFQL	107248.2
0.474	GTR12_HUMAN	Solute carrier family 2, facilitated glucose transporter member 12	W462	DVPAFLKwLSLASLLVYVAAFSIGLGPMPWLVLSEIF	66975.2
0.474	K1468_HUMAN	LisH domain and HEAT repeat-containing protein KIAA1468	W826	EQEGTTGwESLLWVVNQLLPQLIEIVGKINVTSTACV	134647.1
0.474	KCNG1_HUMAN	Potassium voltage-gated channel subfamily G member 1	W278	VESVCVGwFSLEFLLRLIQAPSKFAFLRSPLTLIDLV	57920.1
0.474	LEMD2_HUMAN	LEM domain-containing protein 2	W216	WLSRLLLwASLGLLLVFLGILWVKMGKPSAPQEAEDN	56982.3
0.474	KS6A4_HUMAN	Ribosomal protein S6 kinase alpha-4	W221	GHGKAVDwWSLGILLFELLTGASPFTLEGERNTQAEV	85616.8
0.474	NCKX1_HUMAN	Sodium/potassium/calcium exchanger 1	W584	LDSLIAWwESLLLLLAYAFYVFTMKWNKHIEVWVKEQ	121389.8
0.474	NOX1_HUMAN	NADPH oxidase 1	W9	GNWVVNHwFSVLFLVVWLGLNVFLFVDAFLKYEKADK	64879.1
0.474	MTA2_HUMAN	Metastasis-associated protein MTA2	W297	IRQDFLPwKSLASIVQFYYMWKTTDRYIQQKRLKAAE	75032.6
0.474	PEX12_HUMAN	Peroxisome assembly protein 12	W114	GLPKQQLwKSIMFLVLLPYLKVKLEKLVSSLREEDEY	40802.4
0.474	PONL1_HUMAN	Podocan-like protein 1	W9	AESGLAMwPSLLLLLLLPGPPPVAGLEDAAFPHLGES	56546.6
0.474	ABCA7_HUMAN	ATP-binding cassette sub-family A member 7	W1965	PSARRFLwNSLLAVVREGRSVMLTSHSMEECEALCSR	234380.3
0.474	ADCK2_HUMAN	Uncharacterized aarF domain-containing protein kinase 2	W343	GVLPGIKwLSLPEIVEEFEKLMVQQIDLRYEAQNLEH	68990.7
0.474	BIG2_HUMAN	Brefeldin A-inhibited guanine nucleotide-exchange protein 2 (BIG2)	W1709	SESHREAwTSLLLLLLTKTLKINDEKFKAHASMYYPY	202063.8
0.474	ATG4D_HUMAN	Cysteine protease ATG4D	W297	RPDPTAEwKSVVILVPVRLGGETLNPVYVPCVKELLR	52929.1
0.474	ANR57_HUMAN	Ankyrin repeat domain-containing protein 57	W280	LSASDGKwDSLEGLLTCEPGLLVKRDFITGFTCLHWA	55679.5
0.474	ABCAD_HUMAN	ATP-binding cassette sub-family A member 13	W3796	FPFTASYwKSVGFLVEKRQYFLSSSLFFFNENFDNKG	576231.6
0.474	COX15_HUMAN	Cytochrome c oxidase assembly protein COX15 homolog	W239	VLYCASLwTSLSLLLPPHKLPETHQLLQLRRFAHGTA	46036
0.474	CI004_HUMAN	Uncharacterized protein C9orf4	W63	PRQHPGVwASLLLLLLTGPAACAASPADDGAGPGGRG	37275
0.474	S39AD_HUMAN	Zinc transporter ZIP13	W347	LLEEEDPwRSLQQLLLLCAGIVVMVLFSLFVD (C-term)	39015.9
0.474	SRBP1_HUMAN	Sterol regulatory element-binding protein 1	W876	VDPVAKWwASLTAVVIHWLRRDEEAAERLCPLVEHLP	121691.4
0.474	TLR2_HUMAN	Toll-like receptor 2	W386	NSACEDAwPSLQTLILRQNHLASLEKTGETLLTLKNL	89848.8
0.474	TMCC2_HUMAN	Transmembrane and coiled-coil domains protein 2	W695	LFLLWKHwDSLTYLLEHVLLPS (C-term)	77458.7
0.474	TM178_HUMAN	Transmembrane protein 178	W262	SWSIFCAwCSLGFIVAAGGLCIAYPFISRTKIVHLKS	33022.8

Supplementary Table 3. Search results for putative 1-4-7-8 motifs. The top 100 best scoring matches retrieved by Scansite 3 within the human proteome to a 1-4-7-8 motif search matrix are listed.

Supplementary Table 4

Name	Sequence (5' to 3')
BamH1_RIIalpha_M1	TGGATCCATGAGCCACATCCAGATCCCG
RIIalpha_R45stop_Xho1_Not1	ATAAGAATGCGGCCGCATGTATATCTCCTCGAGTT ATCATGGGGCGCGGGCC
Xho1_SD_6His_AKAP79_M1	ATCGCTCTCGAGGAGATATACATATGCATCATCAC CATCATCATATGGAAACCACAATTTCAGAAATTC
AKAP79_Q427stop_Not1	ATAAGAATGCGGCCGCTCACTGTAGAAGATTGTTT ATTTTATTATCATCAGAG

Supplementary Table 4. Oligonucleotide primer sequences. Primers are listed for constructing a bicistronic vector based on pGEX6P1 containing RIIα (1-45) fused to the C-terminus of GST with AKAP79 bearing an N-terminal 6His tag in the second cistron.
